# Tumeurs bénignes du sein à l’unité de sénologie du Centre Hospitalier Universitaire Aristide Le Dantec de Dakar (Sénégal)

**DOI:** 10.11604/pamj.2017.27.251.12262

**Published:** 2017-08-04

**Authors:** Serigne Modou Kane Gueye, Mamour Gueye, Mariétou Thiam Coulibaly, Diana Mahtouk, Jean Charles Moreau

**Affiliations:** 1 Unité de Sénologie du Centre Hospitalier Universitaire Aristide Le Dantec de Dakar, Sénégal

**Keywords:** Tumeurs bénignes du sein, épidémiologie, traitement, Sénégal, Benign tumors of the breast, epidemiology, treatment, Senegal

## Abstract

**Introduction:**

L'objectif était d'identifier les aspects épidémiologiques, cliniques et thérapeutiques des tumeurs bénignes du sein suivies à l'unité de sénologie du centre hospitalier universitaire Aristide Le Dantec de Dakar.

**Méthodes:**

Il s'agissait d'une étude transversale, descriptive et analytique, portant sur 220 patientes suivies à l'unité de sénologie du Centre Hospitalier Universitaire Aristide Le Dantec de Dakar durant la période allant du 1^er^ janvier 2008 au 31 décembre 2013.

**Résultats:**

Deux cent vingt patientes parmi 984 consultantes présentaient une tumeur bénigne du sein (22,5%). Les tumeurs bénignes du sein représentaient 58,2% de la pathologie tumorale. L'âge moyen était de 24 ans. La tranche d'âge de 11 à 30 ans était la plus représentée soit 70%. La quasi-totalité était en âge d'activité génitale (95%), 58,6% étaient nulligestes. Le motif principal de consultation était une masse mammaire dans 94,5% des cas. Le côté gauche était le plus souvent concerné (49,5%) surtout au quadrant supéro-externe (41,6%). L'échographie était réalisée chez 145 patientes soit 65,9 % des cas. La cytologie retrouvait une hyperplasie épithélio-conjonctive dans la quasi-totalité des cas soit 96,1%. L'histologie réalisée chez 44 femmes confirmait la nature histologique des lésions. L'adénofibrome et les états fibro-kystiques étaient les diagnostics les plus retenus avec respectivement 86,3% et 5,9%. Une tumorectomie a été réalisée chez 28 patientes soit 12,7%, toutes tumeurs confondues. La majorité était suivie sur une durée inférieure à 3 mois avec une évolution favorable de la maladie.

**Conclusion:**

Les tumeurs bénignes du sein sont très fréquentes à en consultation de sénologie. La démarche diagnostique recommandée associe la triade clinico-radio-cytologique et, en cas de doute ou de discordance, une biopsie ou exérèse chirurgicale est incontournable. La prise en charge pas toujours chirurgicale, est fonction de la nature de la tumeur.

## Abstract (en)

**Introduction:**

This study aimed to identify the epidemiological, clinical and therapeutic features of benign tumors of the breast treated in the department of senology at the university hospital Aristide Le Dantec, Dakar.

**Methods:**

We conducted a cross-sectional, descriptive and analytical study of 220 patients treated in the Department of Senology at the University Hospital Aristide Le Dantec, Dakar over the period from 1 January 2008 to 31 December 2013.

**Results:**

220 patients out of 984 consultants had benign tumor of the breast (22.5%). Benign tumors of the breast accounted for 58.2% of tumor pathologies. The average age was 24 years. The age group 11-30 years was the most represented (70%). The quasi-totality of patients were women of childbearing age (95%), 58.6% were nulliparous women. The main reason for consultation was a breast mass in 94.5% of cases. The left side was most often affected (49.5%), especially the upper outer quadrant (41.6%). 145 patients (65.9% of cases) underwent ultrasound. Cytologic examination showed conjunctival epithelial hyperplasia in almost all cases (96.1%). 44 women underwent histologic examination, which confirmed the histologic nature of the lesions. Fibroadenoma and fibrocystic changes were the most retained diagnoses, accounting for 86.3% and 5.9% respectively. 28 patients (12.7%) underwent lumpectomy, all tumor types were taken together. The majority of patients had follow-up appointments within 3 months, with favorable outcome.

**Conclusion:**

Benign tumors are very frequent in senology consultations. The recommended diagnostic approach combines the clinico-radio-cytological triad and, in case of doubt or discrepancy, biopsy or surgical resection are essential. Surgery is not always the treatment of choice. This is based on the nature of the tumor.

## Introduction

Les tumeurs mammaires bénignes sont très courantes en Sénologie. Elles présentent une diversité clinique et pronostique qui justifie toute la variabilité de l'approche thérapeutique. En effet, la plupart de ces tumeurs ont une évolution banale et nécessitent une simple surveillance tandis que pour d'autres tumeurs, la prise en charge est chirurgicale, soit parce qu'elles entraînent des répercussions fonctionnelles gênantes soit parce qu'elles s'accompagnent d'un risque de cancérisation. L'approche thérapeutique globale reste alors dépendante de la nature de la tumeur. Aussi, dans cette étude nous identifions les particularités épidémiologiques, cliniques et thérapeutiques des tumeurs mammaires bénignes suivies à l'unité de sénologie du centre hospitalier universitaire Aristide Le Dantec de Dakar.

## Méthodes

Il s'agissait d'une étude transversale, descriptive et analytique, portant sur 220 patientes suivies à l'unité de sénologie du centre hospitalier universitaire Aristide Le Dantec de Dakar durant la période allant du 1^er^ janvier 2008 au 31 décembre 2013. Etaient incluses toutes patientes chez qui le diagnostic de tumeur bénigne du sein était retenu. Pour chaque patiente, nous avions étudié les caractéristiques socio-démographiques, les signes cliniques et paracliniques de la tumeur mammaire, les traitements reçus et leurs résultats thérapeutiques. L'analyse statistique des données était faite grâce au logiciel SSPSS version 20.0. Les comparaisons uni ou multivariées étaient faites avec le test de Khi2 et les résultats considérés comme significatifs si p < 0,05.

## Résultats

**Fréquence:** Durant la période d'étude, 220 patientes présentaient une tumeur bénigne parmi 984 patientes, soit une fréquence de 22,5%. Dans la pathologie tumorale, les tumeurs bénignes du sein représentaient 58,2% versus 41,8% de cancers.

**Profil socio-démographique:** Les tumeurs bénignes du sein concernaient une population relativement jeune ; d'âge moyen de 24 ans avec des extrêmes de 12 et 67 ans. Cent cinquante-quatre patientes (69,9%) avaient moins de 30 ans (Tableau 1) : la plupart des consultantes venaient de la région de Dakar (36,8%) et de sa banlieue (38,2%), 24,1% des régions et 0,9% des pays limitrophes. Plus de la moitié des patientes étaient célibataires (50,9%) et souvent sans revenu salarial régulier (65%).

**Tableau 1:** Répartition des patientes selon la tranche d’âge

Ages (ans)	Effectifs	(%)
11 - 20	72	32,7
21 - 30	82	37,3
31 - 40	34	15,4
41 - 50	23	10,4
51 - 60	6	2,8
61 - 70	3	1,4
**Total**	220	100

**Antécédents:** L'âge moyen des ménarches était de 14 ans (entre 10 et 19 ans). La notion de ménarches précoces (avant 12 ans) était retrouvée chez 11 patientes (5%). Deux cent neuf patientes étaient en activité génitale (95%). La gestité moyenne était de 2 (entre 1 et 11). Les nulligestes représentaient 58,6% des cas. Au moment du diagnostic, 28 femmes étaient enceintes (12,7%). Soixante-douze patientes (86,8 %) avaient eu leur première grossesse menée à terme avant 30 ans. L'âge moyen au premier enfant était de 21 ans (entre 15 et 37 ans). Les 83 patientes ayant accouché avaient allaité au sein. La durée d'allaitement moyenne était de 18 mois avec des extrêmes de 2 et 24 mois. La notion de contraception était retrouvée chez 33 patientes (15%) dont 63,7 % de contraception hormonale. Huit patientes étaient ménopausées (3,6%). L'âge moyen de la ménopause était de 51 ans (entre 49 et 55 ans ; sans notion de traitement hormonal). Quatorze patientes (6,3%) avaient présenté des antécédents de tumorectomie dont 4 pour fibroadénome. Vingt-trois patientes avaient un antécédent familial de pathologie du sein (10,4%). Il s'agissait d'un cancer dans 13 cas (5,9%).

**Figure 1:**
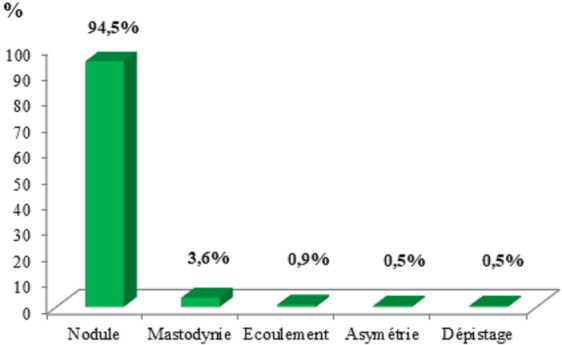
Répartition des principaux motifs de consultation (N = 220)

**Figure 2:**
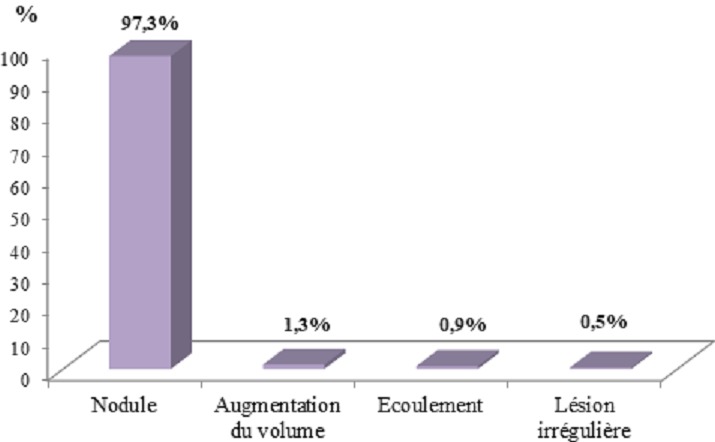
Répartition des anomalies à l’examen clinique (N = 220)

**Aspects cliniques et paracliniques:** Le principal motif de consultation était représenté par le nodule mammaire (94,5%). Elle était associée à une douleur chez 40 patientes (18,2%). La répartition des principaux motifs de consultation est rapportée dans la Figure 1: cent quinze femmes avaient consulté durant la première année d'évolution de la maladie (53 %) avec des extrêmes de 1 semaine et 15 ans (Tableau 2). Pour 3 patientes, ce délai n'était pas précisé. A l'examen clinique, une masse mammaire était retrouvée dans 214 cas, soit 97,3%. La Figure 2 rapporte la répartition des anomalies retrouvées à l'examen : Le côté gauche était le plus atteint (49,5%). L'atteinte était bilatérale dans 8,2 % des cas. Des deux cotés, le quadrant supéro-externe était le plus touché (41,6%). Au plan paraclinique, la mammographie était réalisée chez 36 patientes (16,3%) et retrouvait une anomalie chez 34 d'entre elles (94,5%). Elle était couplée à une échographie chez 21 patientes (58,4%). L'échographie réalisée chez 145 femmes (65,9%) retrouvait une image solide dans 94,5 % des cas et une image kystique dans 5,5% des cas. La classification de l'American College of Radiology (ACR) des résultats radiologiques était précisée dans 84 cas avec 82,2% de classe ACR 1 ou 2 (Tableau 3). La cytoponction mammaire était réalisée chez 206 patientes (93,6%). La cytologie retrouvait une hyperplasie épithélio-conjonctive bénigne chez 198 patientes (96,1%). Pour préciser la nature histologique, une biopsie et/ou une tumorectomie étaient réalisées chez 44 patientes (20%). A l'issue de cette exploration clinique et paraclinique, la pathologie dominante était représentée par le fibroadénome (Tableau 4).

**Tableau 2:** Répartition des délais de consultation

Durées d’évolution (ans)	Effectifs	(%)
≤ 1	115	53
]1 - 3]	66	30,4
]3 - 5]	21	9,7
> 5	15	6,9
**Total**	217	100

**Tableau 3:** Répartition des anomalies radiologiques selon la classification de l’ACR

Classes	Effectifs	(%)
ACR 1	2	2,4
ACR 2	67	79,8
ACR 3	9	10,7
ACR 4	6	7,1
**Total**	84	100

**Tableau 4:** Répartition des diagnostics retenus

Diagnostics	Effectifs	(%)
Fibroadénome	190	86,3
Etat fibro-kystique	13	5,9
Adénose Simple	4	1,8
Adénose Sclérosante	2	0,9
Tumeur phyllode	4	1,8
Lipome	3	1,4
Papillome	2	0,9
Hamartome	1	0,4
Adénome	1	0,4
**Total**	220	100

**Aspects thérapeutiques :** Le traitement médicamenteux reposait sur la progestérone percutanée pour 25 patientes (soit 11,3%) et sur les antalgiques pour 12 patientes (5,4%). Parmi les 220 patientes, une tumorectomie était proposée chez 47 (21,3%) et réalisée chez 28 d'entre elles, soit 12,7% d'actes opératoires. Toutes les patientes avaient bénéficié d'un soutien psychologique. Le Tableau 5 décrit la répartition des indications et actes opératoires selon les affections retrouvées.

**Tableau 5:** Répartition du traitement chirurgical selon l’affection (N = 220)

Affections (N)	Tumorectomies indiquées		Tumorectomies réalisées	
	Effectifs	(%)	Effectifs	(%)
Fibroadénome (190)	32	16,8	17	8,9
Etat fibro-kystique (13)	2	15,4	2	15,4
Tumeur phyllode (4)	4	100	4	100
Adénose Bénigne (4)	2	50	1	25
Adénose Sclérosante (2)	2	100	1	50
Papillome (2)	2	100	2	100
Lipome (3)	3	100	1	33,3
Hamartome (1)	0	0	0	0
Adénome (1)	0	0	0	0

**Surveillance et évolution :** La durée moyenne de suivi était de 3 mois avec des extrêmes de 1 et 48 mois. La majorité des patientes (70%) étaient traitées et suivies sur une durée inférieure à 3 mois. Six patientes (2,8%) étaient suivies sur une durée supérieure à 12 mois. Il s'agissait de 3 cas de fibroadénome, 2 tumeurs phyllodes et 1 cas d'adénose. Aucune complication n'était observée en per ou en post opératoire. A distance, le résultat esthétique et fonctionnel était satisfaisant pour toutes les opérées.

## Discussion

**Epidémiologie des tumeurs bénignes du sein:** Dans notre série, la fréquence de tumeurs bénignes du sein (58,2%) est plus élevée que les 45,8% rapportés par Lutula au Mali en 2008 [1]. Ailleurs en Guinée, Diallo en 1996 trouvait une fréquence de 63,2% [2]. Ces chiffres témoignent de la fréquence relativement élevée des tumeurs bénignes du sein en Afrique subsaharienne. Dans ce contexte, la population concernée est relativement jeune ; et les tumeurs bénignes du sein reste l'apanage des adolescentes et des femmes en activité génitale [3, 4]. La provenance des patientes est très variable et reste dépendante de l'accessibilité des structures de soins mammaires: 75% du milieu urbain (Dakar et sa banlieue) tandis que Diallo en Guinée trouvait 85,5% du milieu rural avec comme constante un niveau de revenu faible et un statut matrimonial dominé par les célibataires [1, 2]. Vu la diversité histologique des tumeurs bénignes du sein, les facteurs de risque sont difficiles à cerner mais restent intimement liés à la nature de chaque affection en cause.

**Approche diagnostique :** Devant une tumeur bénigne du sein, il convient d'être très rigoureux quant à la démarche à adopter. L'interrogatoire et l'examen physique soigneusement menés restent obligatoires avant d'avoir recours à des examens complémentaires radiologiques ou anatomo-pathologiques. Plusieurs motifs de consultation peuvent être retrouvés (nodule du sein, écoulement mamelonnaire, adénopathie axillaire, modification cutanée, etc). Dans notre série et celle de Foko au Mali [5], le nodule était le principal motif de consultation avec respectivement 94,5% et 91,9%. La moitié des patientes (53%) avait consulté dans la première année d'évolution de la maladie ; Lutula avait trouvé un peu moins (44,6%) [1]. Cela témoigne des efforts considérables de sensibilisation et de dépistage qui restent à faire dans des pays en développement afin de réduire les délais de consultation. La survenue de tumeurs bénignes ne semble pas liée à la latéralité; dans 49,5% des cas, la tumeur a intéressé le sein gauche. Lutula avait rapporté 68,5% de localisation gauche alors que selon Foko [5], l'atteinte prédominait au sein droit (50,8%). Le quadrant supéro-externe est le siège de prédilection des tumeurs mammaires bénignes ; 41,6% dans notre série et 40,6% dans celle de Lutula. Dans la démarche diagnostique, même si certains aspects cliniques et radiologiques sont très évocateurs, le diagnostic doit être confirmé par l'anatomie pathologique (cytologie ou histologie). La cytologie peut être suffisante (en cas de triade cyto-radio-clinique concordante) si pratiquée par un opérateur expérimenté, mais en cas de doute il faut recourir à l'histologie. Plusieurs auteurs sont pour une confirmation histologique d'emblée et pour toutes [1, 2, 5]. Dans notre série, nous avons eu recours à la cytologie pour 206patientes (93,6%) et pour 44 cas douteux, discordants ou dans un contexte à risque (20%), il a fallu aller jusqu'à l'histologie. La pathologie tumorale bénigne est dominée par le fibroadénome et la maladie kystique [6]. Le même constat est fait par Lutula [1] au Mali avec 33,3% pour le fibroadénome et 27,8% pour les états fibrokystiques ; tandis que Diallo, en Guinée, rapporte 53,6% de fibroadénome [2].

**Aspects thérapeutiques :** Devant les tumeurs bénignes du sein, les indications thérapeutiques sont fonction de la nature de la lésion. Une sanction chirurgicale est de règle pour certaines tumeurs mammaires bénignes tandis que pour d'autres, l'abstention avec une surveillance peut suffire. Parfois un traitement médicamenteux peut s'avérer nécessaire. Dans notre étude, nous avons eu recours à ce type de traitement pour 37 patientes (16,8 %) dont 25 traitées par progestérone percutanée et 12 par antalgiques. Notre pourcentage de tumorectomie est relativement faible (12,7%) contrairement à celui de Lutula [1] qui la réalise dans 66,7% des cas. En fait, les indications chirurgicales restent dépendantes de la nature de la tumeur. Dans le fibroadénome, aucun traitement médical efficace n'a été démontré; la place de la chirurgie reste limitée. Une décision d'exérèse chirurgicale pourra être prise soit d'emblée pour une taille supérieure à 3 cm, un âge supérieur à 35 ans, des antécédents personnels ou familiaux de cancer du sein [7] . Dans notre série, l'indication chirurgicale a été posée dans 16,8% des cas; le plus souvent pour des raisons de gros volume et pour des raisons liées à l'âge supérieur à 40 ans. En effet, nous décidons d'être le moins interventionniste dans la mesure où la surveillance s'applique bien à cette affection qui rentre dans le Concept des « Aberrations of Normal Development and Involution » (ANDI) [7]. Dans les états fibrokystiques, les traitements médicamenteux symptomatiques, les mesures promotionnelles et la surveillance sont toujours de mise [8]. La place de la chirurgie est encore plus limitée. Dans notre pratique, l'abstention chirurgicale (77%) et la surveillance, avec au besoin un traitement symptomatique (15,4%), occupent une place importante dans le traitement des états fibrokystiques.

Concernant les tumeurs phyllodes avant 20 ans, selon Hugues et Mansel, elles peuvent être traitées par énucléation, car elles se comportent presque toujours comme un fibroadénome [8]. La situation est moins claire chez les patientes plus âgées ; peu de chirurgiens ont rapporté une large expérience pour être formels sur la meilleure attitude. Pour notre part, le traitement des tumeurs phyllodes doit rester chirurgical. Les tumeurs de grade 1 et 2 sont traitées par exérèse complète emportant une collerette de tissu sain de 10 mm. Pour les tumeurs phyllodes de grade 3, le traitement de référence reste la mastectomie simple, sans curage axillaire ; une radiothérapie ou une chimiothérapie peuvent également être discutées en réunion de concertation pluridiciplinaire [9]. Haagensen rapporte un taux de récidive locale de 28% chez les 43 patientes traitées par exérèse locale avec un recul de 10 ans [10]. Le traitement des autres lésions bénignes dépend de leur nature histologique, de la taille, des antécédents, des anomalies fonctionnelles associées et du désir de la patiente. La chirurgie est systématique devant, une adénose sclérosante, un papillome périphérique et en cas d'association à une lésion atypique ou in situ [11–13]. Tout papillome qui présente des atypies doit bénéficier d'une sanction chirurgicale (pyramidectomie) en raison du haut risque de cancer associé [14, 15]. Il n'y a pas de consensus quant à la prise en charge des papillomes sans atypie [16, 17]. Les 2 cas rapportés dans notre série ont bénéficié d'une pyramidectomie ayant confirmé le diagnostic de papillome sans atypies.

## Conclusion

Les tumeurs bénignes du sein sont très fréquentes à l'unité de sénologie du centre hospitalier universitaire Aristide Le Dantec de Dakar. L'échographie reste l'examen d'imagerie de référence devant une masse mammaire chez la femme jeune. La cytologie reste un examen incontournable dans le diagnostic. Lorsque le trépied « clinique - échographie - cytologie » est concordant, le diagnostic peut être confirmé. La prise en charge est fonction de la nature de la tumeur pouvant aller de la simple surveillance à la tumorectomie: tout n'est pas à opérer.

### Etat des connaissances actuelle sur le sujet

- La fréquence des tumeurs bénignes par rapport aux cancers du sein;
- Les Signes cliniques et paracliniques des tumeurs bénignes du sein;
- Les moyens radiologiques et cyto-histogiques du diagnostic.

### Contribution de notre étude à la connaissance

- Précisions sur les indications thérapeutiques;
- Les résultats d'un traitement moins chirurgical;
- L'expérience de l'Unité de Sénologie du CHU de Dakar.

## Conflits d’intérêts

Les auteurs ne déclarent aucun conflit d'intérêts.
